# Preference Alters Consumptive Effects of Predators: Top-Down Effects of a Native Crab on a System of Native and Introduced Prey

**DOI:** 10.1371/journal.pone.0051322

**Published:** 2012-12-06

**Authors:** Emily W. Grason, Benjamin G. Miner

**Affiliations:** 1 Western Washington University, Biology Department, Bellingham, Washington, United States of America; 2 Shannon Point Marine Center, Anacortes, Washington, United States of America; University of Fribourg, Switzerland

## Abstract

Top-down effects of predators in systems depend on the rate at which predators consume prey, and on predator preferences among available prey. In invaded communities, these parameters might be difficult to predict because ecological relationships are typically evolutionarily novel. We examined feeding rates and preferences of a crab native to the Pacific Northwest, *Cancer productus*, among four prey items: two invasive species of oyster drill (the marine whelks *Urosalpinx cinerea* and *Ocenebra inornata*) and two species of oyster (*Crassostrea gigas* and *Ostrea lurida*) that are also consumed by *U. cinerea* and *O. inornata*. This system is also characterized by intraguild predation because crabs are predators of drills and compete with them for prey (oysters). When only the oysters were offered, crabs did not express a preference and consumed approximately 9 juvenile oysters crab^−1^ day^−1^. We then tested whether crabs preferred adult drills of either *U. cinerea* or *O. inornata*, or juvenile oysters (*C. gigas*). While crabs consumed drills and oysters at approximately the same rate when only one type of prey was offered, they expressed a strong preference for juvenile oysters over drills when they were allowed to choose among the three prey items. This preference for oysters might negate the positive indirect effects that crabs have on oysters by crabs consuming drills (trophic cascade) because crabs have a large negative direct effect on oysters when crabs, oysters, and drills co-occur.

## Introduction

Predation is a major force structuring un-invaded [1], [2] and invaded [3], [4] communities. Directly, predators can limit [5], [6], regulate [7], or extirpate [8] prey populations through consumption. These effects can, in turn, be propagated indirectly through the community in many ways, including trophic cascades, indirect facilitation, and apparent competition [9]. Direct and indirect effects of predation can engender quantitative, as well as qualitative, shifts in community composition. For instance, predators can enhance community diversity by consuming competitively dominant prey, thereby reducing interspecific competition and facilitating the persistence of competitively inferior prey [10]. The population and community effects of predators depend on the number of prey they consume, and their preference.

The trophic dynamics of an invaded food web are not always predictable because predator-prey interactions are typically evolutionarily novel. Predators might not be able to recognize an invasive prey as potential food, or overcome defenses of invasive prey [11]. Even where predators are generalists and invasive prey are ecologically similar to natives, an invaded trophic chain might not function in the same way as a comparable chain comprised only of co-evolved natives [12]. Prey preference in the European green crab, *Carcinus maenas*, destabilized competitive interactions between native and non-native clams, allowing the established non-native gem clam, *Gemma gemma*, to become invasive [13]. Appropriate management of invasions therefore requires investigation of the pathways by which predators impact the community.

**Figure 1:**
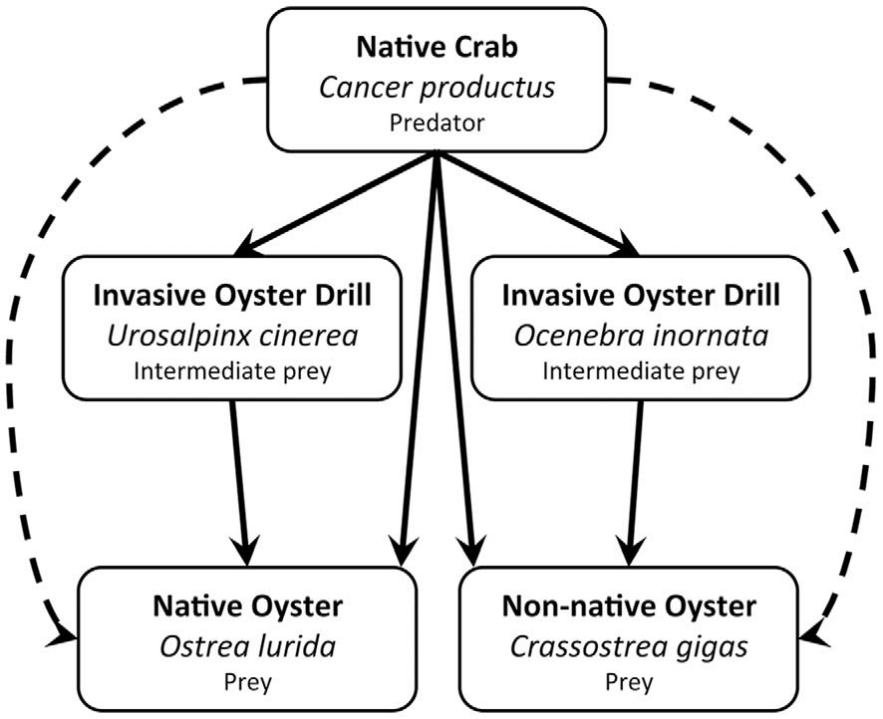
Diagram of trophic interactions in our study system. Diagram of trophic interactions among native red rock crabs, *Cancer productus*, two species of invasive oyster drill, *Urosalpinx cinerea* and *Ocenebra inornata*, and two oyster species, native *Ostrea lurida* and non-native *Crassostrea gigas*. Solid lines represent direct interactions, dashed lines indirect interactions with all arrows pointing in the direction of the trophic effect.

We investigated a commercially and ecologically important food web with a native predator, mediated by invasive intermediate prey (Figure 1). In Washington State, two invasive species of oyster drill, the marine whelks *Ocenebra inornata* and *Urosalpinx cinerea*, are common predators of oysters. Because drills threaten the recovery efforts of a rare native oyster (*Ostrea lurida*) and yield of commercial oyster harvest, the effects of drills as predators of oysters have been well studied [14]–[16]. Drills of both species generally prefer to prey on small oysters, and can consume juveniles at a rate of 0.3 oysters per day [16] potentially limiting oyster populations at this life history stage. However, relatively little is known about predatory control that native crabs, which are common predators of marine snails, exert on this system (but see [12]). As predators of both drills and oysters, crabs could impact oysters directly by preying on them, and indirectly by consuming drills and releasing oysters from drill predation (Figure 1). Where all three species coincide, the relative importance of direct and indirect effects of crabs on oysters will be determined by which prey, drills or oysters, the crabs prefer. The drills’ habitat overlaps with that of several native decapod crabs, including the red rock crab, *Cancer productus* [17]. A large-clawed generalist predator, *C. productus* preys on native whelks and can strongly influence the community structure of intertidal habitats [18].

This system is also characterized by asymmetric intraguild predation (IGP): crabs are both predators of, and competitors with, drills, but drills do not prey on crabs [19]. Similar systems are common in native intertidal communities where generalist crabs and seastars feed on both predatory snails and bivalve prey of snails (e.g., [20], [21]). Additionally, models predict unintuitive dynamics caused by IGP in systems where the intraguild predator is a non-native species [22], and demonstrate that IGP by non-natives can accelerate the speed of invasion [23]. Less is known about IGP in systems where the intraguild prey is a non-native. Therefore, to estimate the potential for both direct and indirect effects of crabs on oysters, and the dynamics of IGP, we investigated crab feeding rates and preferences for invasive drills, and two species of oyster. This information will be critical to predicting the trajectory of this system, and will help enhance our understanding of the role of IGP in invaded communities.

## Materials and Methods

In two experiments conducted at Shannon Point Marine Center, in Anacortes, WA, we estimated feeding rates and preferences of the native red rock crab, *Cancer productus*, for two species of juvenile oysters (*Crassostrea gigas*, and *Ostrea lurida*) and their invasive drill predators (*Urosalpinx cinerea*, and *Ocenebra inornata*). We initially tested whether the rates of consumption and preference of crabs differed for the two oyster species. We then investigated crab feeding on both species of invasive drill and non-native *C. gigas*.

### System

Both Atlantic (*U. cinerea*) and Japanese oyster drills (*O. inornata*, synonyms include *Ceratostoma inornatum*, and *Ocinebrellus inornatus*) were unintentionally introduced to the Pacific Northwest by the early 1920’s [24]. Both drills arrived as hitchhikers on cultch (newly settled larvae on shell material) of non-native oysters imported to buoy the oyster-growing industry – *U. cinerea* from the east coast of the United States on *Crassostrea virginica* (Eastern oyster), and *O. inornata* from the Asian Pacific Ocean on *Crassostrea gigas* (Pacific oyster). Because they lack a larval planktonic stage, drills are dispersed primarily through human-mediated transport. Notably, native whelks are not considered a pest in oyster beds [24], so we did not include them in our study.

While at least five species of oyster are currently grown and harvested in Washington, we were particularly interested in the effects of crabs on non-native *C. gigas* and native *O. lurida*. The Pacific oyster, *C. gigas*, is the most widely introduced, and commercially valuable oyster species worldwide, and has also established naturally recruiting, wild populations in bays and inland waters of Puget Sound and coastal Washington [25]. In contrast, populations of *O. lurida*, the only native oyster species in Washington, collapsed in the late 1800’s due to overharvesting and pollution, and have not sustained a harvestable wild population since [26]. Drills are pests to the oyster industry, costing shellfish growers money in control and eradication efforts, as well as in decreased revenues. Additionally, drills could be inhibiting recovery efforts aimed at restoring wild populations of the rare and ecologically important native *O. lurida* [16], [27].

Due to their relatively thin shells, juveniles of both oyster species are most vulnerable to drill predation [16]. Both drills consume small individuals of *C. gigas* and *O. lurida* at similar rates of about 0.25–0.30 oysters per drill per day [16]. Preference for the two species of drills between these two species of oyster has already been established and so was not tested here. Despite divergent evolutionary histories with these oyster prey, both drills prefer *C. gigas* to *O. lurida* oysters of similar size [16]. Population growth of oysters depends on at least some recruits reaching reproductive size without being consumed. Survival of oyster populations, therefore, could be strongly affected by predation on juvenile oysters by adult drills (E. Grason, unpublished data). Buhle and Ruesink [16] hypothesized that crab predation might limit distribution of drills, and therefore their effects on oysters, but there is, as yet, no experimental support for this hypothesis.

### Animal Collection and Husbandry

Both male and female red rock crabs, *C. productus*, were collected by hand and trap from beaches and docks around Anacortes, WA. We assumed crabs had no experience with either species of drill or oyster because those organisms or their remains were not found in the areas where crabs were collected. Neither oysters nor invasive drills were present at these sites, likely because oysters have not historically been cultured at these sites. This reduces the probability that crabs had prior experience with any of the species of prey used in our experiments and allows us to infer that preferences are innate. While in the lab, crabs were maintained in flow-through aquaria, on a diet of mussels (*Mytilus* sp.) and frozen fish fillet (*Tilapia* sp.). Crabs were not starved prior to the feeding experiments.

Atlantic drills, *U. cinerea*, were collected from naturally recruiting *C. gigas* reefs in the southeastern corner of Willapa Bay, WA. Japanese drills, *O. inornata*, were collected from commercial *C. gigas* beds owned by Taylor Shellfish Farms in West Samish Bay, WA. Drill species were maintained in separate, closed 140 L aquaria, and allowed to feed *ad libitum* on mussels and *C. gigas* juveniles.

Juvenile oysters of both species were obtained as seed (singles) from Taylor Shellfish Farm hatcheries. At the time of experiments, oysters were of a size at which they could typically be out-planted by commercial or recreational growers (*C. gigas = *2.7±0.4 cm, *O. lurida = *2.5±0.2 cm). Oysters were held in sea-tables and had access to a limited amount of plankton that came in with the natural seawater. We supplemented this diet with commercial shellfish diet (Shellfish Diet 1800-Reed Mariculture) at least once a week.

### Preference Experiments

For the purpose of this study, preference was defined as a deviation of feeding behavior (proportion of prey consumed of one type) in the presence of choice compared to feeding behavior without choice [28]. Therefore each experiment included treatments in which crabs were offered one prey type only, and one treatment where all prey types were offered simultaneously in equal abundance. This design has the advantage of providing researchers with several relevant estimates of feeding rate and clearly differentiates between preference and electivity (for a discussion of “preference” versus “electivity” see [29]). Electivity can cause diet changes in a predator via “prey switching”, which occurs when predators attack different prey depending on relative prey abundance [30]. Such “switching” behavior can be ecologically relevant, but was not tested in this experiment, as we chose to focus on preference as a trait of crabs, rather than how contexts affect the interaction between crabs and the two types of prey.

Two experiments were conducted to estimate preference and feeding rates among the prey items. We first estimated *C. productus* feeding rates on and preferences among juvenile oysters, Pacific (*C. gigas*) and Olympia (*O. lurida*) oysters (September, 2009). Then, we estimated *C. productus* feeding rates on and preferences for juvenile oysters, (*C. gigas*) and both species of drill (*U. cinerea* and *O. inornata*) (October, 2009). Conducting these experiments separately, rather than as a single experiment with all four prey types, allowed for greater replication. In this way we improved resolution in determining crab preference between the two species of oyster because we thought that a general preference for oysters over drills might obscure any difference between the two oyster species. Comparing preference among adult drills and juvenile oysters was ecologically relevant to oyster restoration efforts as well as aquaculture scenarios. Oysters in both experiments were marked with enamel to facilitate correct species identification. Different individual crabs were used in each of the experiments. In the first experiment, crabs (mean carapace width ± SE: 106.7±2.1 mm) were randomly assigned to one of three treatments (n = 12): (1) 25 *C. gigas* only, (2) 25 *O. lurida* only, or (3) 25 *C. gigas* and 25 *O. lurida*. In the second experiment, crabs (mean carapace width ± SE: 107.2±2.2 mm) were randomly assigned to one of four treatments (n = 14): (1) 25 *C. gigas* only, (2) 25 *O. inornata* only, (3) 25 *U. cinerea* only, or (4) 25 *C. gigas*, 25 *O. inornata*, and 25 *U. cinerea*. We used only *C. gigas* in this experiment because there was no preference between oyster species in our first experiment and this species is more readily available and not of conservation concern. Observation confirmed that there was no predation by drills on oysters during this experiment, and all oyster mortality was due to crab predation.

One day prior to each experiment, individual crabs were placed in separate flow-through bins with 10 individuals of their assigned prey types to allow feeding behavior to stabilize [31]. At the start of the experiment, we removed all waste, uneaten food, and shell material from the bins, and added individuals of each prey type appropriate for the treatment of each bin. The number of surviving prey of each type was recorded at 24 hours without replacement. In one replicate, a crab consumed all available prey of one type (all individuals of *C. gigas* consumed by one crab in choice treatment of oyster preference experiment).

### Analysis

To determine whether crabs preferred one type of prey, we developed a new method, which is described below. Currently there is debate about how best to statistically test for preference, and the proposed methods all have benefits and drawbacks [28], [32]–[35]. With all available methods, it is difficult to test for preference with more than two species. Because we wanted to test for preference for more than two prey species, we developed an alternative method carefully considering the concerns raised in previous studies [26], [30]–[33].

To test for preference, we used the interaction term of a two-factor ANOVA with prey type and choice both modeled as fixed factors. Prey type had two levels in the first experiment (two species of oyster) and three levels in the second experiment (two species of drill and one species of oyster), and choice had two levels in both experiments (whether crabs had a choice of prey or not). The response variable, the proportion of prey consumed, was calculated as follows. In the no-choice bins, we randomly grouped one replicate from each prey type together and calculated the proportion of prey consumed for each prey type out of the total prey consumed for the group. In the choice treatment, we calculated the proportion of prey consumed for each prey type out of the total prey consumed in a single replicate bin. If the proportion of each prey type consumed was different when crabs were offered a choice, versus when crabs were only offered a single type, the interaction term of a two-factor ANOVA with prey type and choice as fixed factors would be significant. However, because of our calculations of the response variable, the data were not independent – the proportions of each prey type consumed in each replicate where crabs were allowed a choice were, by definition, constrained to total to 1.0. Therefore, use of the F-statistic distribution would calculate an incorrect probability of type 1 error. We therefore generated a null distribution of F-ratios for the interaction between choice and prey type by randomly assigning prey types to bins in both the no choice and choice treatments for each experiment (10,000 iterations). We used the generated null distribution to calculate the probability of a type I error for the observed F-ratio for the interaction between choice and prey type.

To ensure that the results were not particular to a random pairing of bins in the no choice treatment, we randomly re-paired bins in the no choice treatment and re-ran the analysis 1,000 times. Distributions of *P* values of the interaction between choice and prey type were generated and compared to an alpha value of 0.05 to determine whether crabs preferentially fed on the prey species. All analyses were conducted in R [36].

## Results

### Oyster Preference Experiment

**Figure 2:**
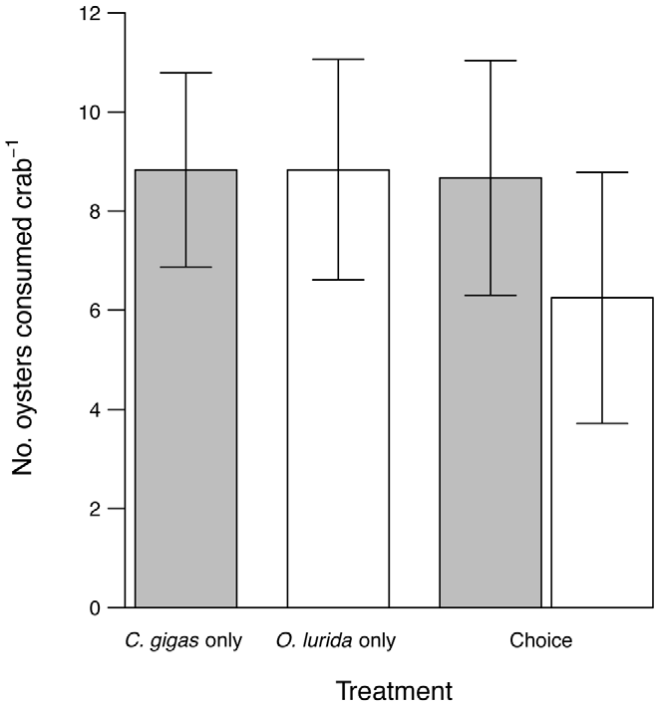
Crab feeding rates and preference on native and non-native oysters. Number (mean ± SE) of oysters consumed by crabs over 24 hours in the oyster preference experiment. Gray bars are *Crassostrea gigas*, Pacific oysters, and white bars are *Ostrea lurida*, Olympia oysters. Crabs were randomly assigned to one of three treatments (n = 12): (1) 25 *C. gigas* only, (2) 25 *O. lurida* only, or (3) the treatment labeled “Choice”, 25 *C. gigas* and 25 *O. lurida*.

There was no evidence that crabs preferred one species of juvenile oyster to the other (Figure 2). Crabs consumed a similar number of *C. gigas* as juvenile *O. lurida* when they were offered only one species and denied a choice, but consumed slightly more *C. gigas* than *O. lurida* when offered a choice. However, all of the *P* values of the interaction between prey type and choice generated in the random pairing of bins were greater than 0.05, suggesting that the interaction term is not significant. Crabs, therefore, do not consume a different proportion of juvenile *C. gigas* and *O. lurida* in the presence and absence of choice.

### Pacific Oysters and Drills Preference Experiment

**Figure 3:**
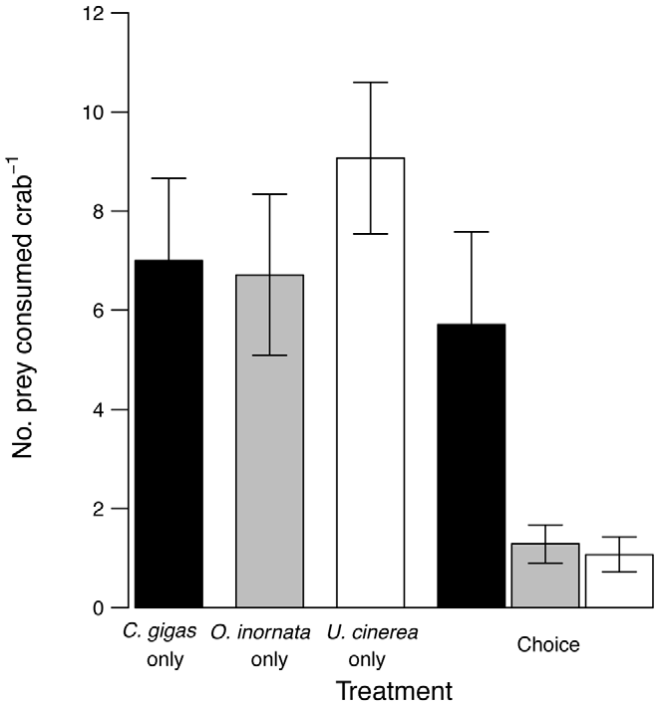
Crab feeding rates and preference on oyster drills and *Crassostrea gigas.* Number (mean ± SE) of prey consumed by crabs over 24 hours in the drill/oyster preference experiment. Black bars are *Crassostrea gigas*, Pacific oysters, gray bars are *Ocenebra inornata*, Japanese drills, and white bars are *Urosalpinx cinerea*, Atlantic drills. Crabs were randomly assigned to one of four treatments (n = 14): (1) 25 *C. gigas* only, (2) 25 *Ocenebra inornata* only, (3) 25 *Urosalpinx cinerea* only, or (4) the treatment labeled “Choice”, 25 *C. gigas*, 25 *O. inornata*, and 25 *U. cinerea*.

There was evidence that crabs preferred non-native oysters to drills (Figure 3). When offered only one type of prey, crabs did not consume prey types at different rates. An average of 7 juvenile *C. gigas*, 7 *O. inornata*, or 9 *U. cinerea* were consumed per crab per day in the absence of choice. However, in treatments in which crabs were allowed to choose from among the three prey types, oysters were disproportionately preyed on compared to drills–crabs consumed nearly 6 times as many oysters as either species of drill. Approximately 99.9% of the *P* values of the interaction between factors prey species and choice generated by randomly pairing bins were less than 0.05, indicating that crabs consumed a significantly different proportion of oysters in the presence and absence of choice.

## Discussion

Whether or not crabs demonstrated a preference among prey choices depended on the types of prey offered. In the first experiment, when both prey were oysters, the predator expressed no preference for either the native or the invasive oyster. In the second experiment, when both species of drill were offered along with one species of oyster, crabs strongly preferred *C. gigas,* oysters, to either species of drill. This difference is likely not a product of different handling times for each prey species, as all prey types were consumed at relatively similar rates when offered in single species treatments. It is probable that the preference for *C. gigas* results from a relatively greater energy yield per unit effort required to obtain the food from oysters as opposed to well-armored drills.

At sites where crabs, drills, and oysters co-occur, crab preference could cause a direct negative effect on juvenile oysters that negates the positive indirect effect of crabs eating drills. In our treatment that allowed crabs to choose, crabs consumed an average of about 6 oysters and 2 drills (one of each species) per day. Individual drills consume at most approximately 0.3 juvenile oysters per day [16]. In the presence of choice, therefore, the direct negative effect of an individual crab on oysters is approximately -6 oysters per day, while the positive indirect effect is +0.6 oysters per day. The net effect of crabs in the system is still strongly negative: 5.4 oysters removed per crab per day. Therefore, despite the fact that they can be highly efficient and motivated predators on drills, *C. productus* is likely to exert stronger direct consumptive effects on oysters. Long-term dynamics in this system will depend on population responses of oysters to predation by both crabs and drills. For instance, predation on oysters could facilitate a population increase in crabs that could then impact drills negatively via apparent competition, or deplete oysters to the extent that crabs switch their search image to prey primarily on drills.

As an intraguild predator, therefore, *C. productus* interacts more strongly with drills as a competitor for oysters than as a predator. It is notable that this crab-drill-oyster system does not have the characteristics of systems in which IGP is believed to be stabilizing. In asymmetric IGP systems, a condition for stability is that the intraguild predator is the stronger competitor for the extraguild resource [37]. In this study, not only is crab-drill predation unidirectional, but it also seems likely, based on differences in per capita feeding rates, that crabs are better at exploiting oysters than drills. Other researchers have pointed out that models of IGP stability have required systems of closed populations, a condition which is clearly not met in many marine habitats where species often have widely-dispersing pelagic larvae [21]. Thus the population growth rate of oysters in our system might be decoupled from local community and population dynamics. Alternatively, theory predicts that the three species might be able to co-exist exist in habitats where alternative food sources not important in the crab’s diet (e.g., barnacles) could sustain drill populations [38].

Our laboratory experiments did not account for spatial and temporal heterogeneity that will affect the strength of these interactions in the field. We purposely eliminated structure in our mesocosms to prevent drills from using refugia to avoid predation, because both drill species reduce feeding and increase use of refugia when they detect chemicals released by *C. productus* consuming conspecific drills [39]. This design enabled us to better isolate consumptive (predation) from non-consumptive (intimidation) effects of crabs in this system. However, in the field, we would expect that structural complexity could further reduce the rate at which crabs are able to prey on drills, and would also reduce the rate at which feeding drills consume oysters. Therefore, our estimates are likely conservative, as such non-consumptive effects would only increase the relative importance of the direct effects of crab predation on oysters.

Notwithstanding the evidence presented here that crabs are unlikely to reduce drill densities in oyster beds, other researchers have noticed that in Willapa Bay, WA, there is an overall negative correlation between abundance of crabs and oyster drills [17]. This might be explained by the fact that crabs do still prey on drills at low levels, even when oysters are present. Additionally, along with emigration and predation, inducible defenses of drills in response to crabs likely carry fitness costs that explain lower drill densities where *C. productus* is present.

Our study suggests the interesting possibility that oysters facilitated the invasion of both species of drill, not only as a vector (non-native oysters) and food source (both native and non-native oysters), but also by reducing the potential for biotic resistance by native crabs. Both drills were originally introduced simultaneously with oysters, and, notably, are almost entirely restricted to oyster beds in Washington State. This granted drills a degree of enemy release, at least in the short term, because crabs preferentially prey on oysters when they have a choice. The corollary to this idea is that while *C. productus* might not strongly affect drill populations in oyster beds, crabs could help limit the range of invasive drills to oyster beds. Where oysters are rare, it is possible that crabs will switch to consuming relatively more drills, and crabs could thereby provide greater biotic resistance against drill incursion into these habitats. This provides one way that context-dependent species interactions, such as those mediated by preference, could be particularly important in invaded systems.
